# miR-29b and miR-29c Are Involved in Toll-Like Receptor Control of Glucocorticoid-Induced Apoptosis in Human Plasmacytoid Dendritic Cells

**DOI:** 10.1371/journal.pone.0069926

**Published:** 2013-07-23

**Authors:** Yongfeng Hong, Jianxian Wu, Jingpu Zhao, Huiping Wang, Yi Liu, Tianping Chen, Xiuli Kan, Qianshan Tao, Xianshan Shen, Kaili Yan, Zhimin Zhai

**Affiliations:** 1 Department of Rehabilitation, The Second Affiliated Hospital to Anhui Medical University, Economic Development Zone, Hefei City, Anhui, China; 2 Hematology Department and the Blood Research Center, The Second Affiliated Hospital to Anhui Medical University, Economic Development Zone, Hefei City, Anhui, China; Philipps University, Germany

## Abstract

Glucocorticoids (GCs) are frequently used to treat many of the acute disease manifestations associated with inflammatory and autoimmune disorders. However, Toll-like receptor (TLR) pathway-activated plasmacytoid dendritic cells (pDCs) are resistant to GC-induced apoptosis, which leads to the inefficiency of GCs in the treatment of type I interferon-related autoimmune diseases, such as systemic lupus erythematosus (SLE). Therefore, compounds promoting pDC apoptosis may be helpful for improving the efficacy of GCs. In this study, we performed screening to identify microRNAs (miRNAs) involved in TLR-inhibited GC-induced pDC apoptosis and found an array of miRNAs that may regulate pDC apoptosis. Among those demonstrating altered expression, 6 miRNAs were inhibited in TLR-activated pDCs. Bioinformatics analysis and functional studies indicated that miR-29b and miR-29c were 2 key miRNAs involved in TLR-inhibited GC-induced pDC apoptosis. Furthermore, both of these miRNAs promoted pDC apoptosis by directly targeting Mcl-1 and Bcl-2 in human primary pDCs. Our findings provide new targets that could improve the efficacy of GCs for the treatment of SLE.

## Introduction

Glucocorticoids (GCs) are small lipophilic compounds that mediate a plethora of biological effects by binding the intracellular glucocorticoid receptor (GR), which, in turn, translocates to the nucleus and directly or indirectly regulates gene transcription [1]. Dexamethasone (Dex) is a potent synthetic member of the GC class of steroid drugs that acts as an anti-inflammatory and immunosuppressant. GCs also have inhibitory effects on a broad range of specific immune responses mediated by T cells and B cells and potent suppressive effects on the effector functions of phagocytes. In addition, GCs have been shown to affect the viability of dendritic cells (DCs), to selectively down-regulate the expression of co-stimulatory molecules on viable DCs, and strongly reduce the immunostimulatory properties of DCs both in vitro and in vivo [2], [3]. Because of their inhibitory effects on both acquired and innate immunological functions, GCs are remarkably efficacious in managing many of the acute disease manifestations of inflammatory and autoimmune disorders [1]–[3].

Plasmacytoid dendritic cells (pDCs) are a distinct DC subtype that specializes in the rapid production of large amounts of type I interferon (IFN) in response to viral stimulation. In contrast to other DCs, pDCs selectively express Toll-like receptor (TLR)7 and TLR9, which sense non-self-nucleic acids during microbial infection [4]. pDC deficiency leads to reduced IFN-α production, which results in an inadequate immune response and susceptibility to viral infection. Alternatively, overexpression of IFN-α can induce hyper-immune activation, which may lead to autoimmune conditions [5]. Type I IFN is considered to be critical for systemic lupus erythematosus (SLE) disease pathogenesis and the increased expression of IFN-regulated genes (termed the IFN signature), and the levels of type I IFN correlate with the production of autoantibodies and disease activity. In lupus, GCs are typically administered orally on a daily basis because every-other-day regimens cannot adequately control disease. When doses greater than 40 mg per day are required, patients receive intravenous methylprednisolone (Solu-Medrol) pulse therapy. Although such treatment can transiently reduce disease activity, it rarely induces remission or prevents organ damage [6]. In addition, it was shown that anti-apoptotic pDCs in SLE patients are the major source of type I IFN and that blocking pDC function greatly improves the anti-inflammatory effects of GC drugs [6]. Further studies indicated that this anti-apoptotic effect was dependent on TLR-induced autocrine TNF-α and IFN-α, which serve to increase the expression ratio of anti-apoptotic (Bcl-2, Bcl-xL, BIRC3, CFLAR) to pro-apoptotic genes (Caspase-8, BID, BAD, BAX) [7]. Therefore, drugs that inhibit TLR-induced anti-apoptotic pDCs may enhance the efficacy of GC treatment for autoimmune diseases.

MicroRNAs (miRNAs) are small RNA molecules that function in the post-transcriptional regulation of gene expression, and the deregulation of miRNAs is associated with various diseases [8]. Many cancer-related miRNAs have been identified as oncogenic and apoptotic proteins, such as p53, p21, c-Myc, and Bcl-1 gene family members, are regulated by different miRNAs in different diseases [9]. Notably, the miR-17-92 cluster accelerates c-Myc-induced lymphoma development, leading to lymphoproliferative disease and autoimmunity by directly targeting the tumor suppressor PTEN and the pro-apoptotic protein Bim [10]. Therefore, miRNAs are likely also involved in TLR-inhibited pDC apoptosis during GC treatment, and identifying these miRNAs will provide new targets for pDC-related autoimmune diseases and improve the efficacy of GC drugs for the control auto-inflammatory diseases.

In this study, we compared human primary pDC miRNA profiles obtained under different conditions. A total of 36 GC-induced miRNAs were inhibited by TLR stimulation, whereas 20 GC-inhibited miRNAs were induced by TLR activation. Further analysis showed that 6 miRNAs were predicted to target Bcl-2 family members, and functional screening demonstrated that miR-29b and miR-29c were involved in TLR-inhibited Dex-induced pDC apoptosis. Therefore, in addition to its previously proposed role as a TLR7/9 antagonist, miR-29 could also represent a new target for anti-autoimmune drug discovery.

## Methods

### Ethics Statement

All participants signed a statement of written informed consent. The procedures described in this study were approved by the ethics committee of the Second Affiliated Hospital to Anhui Medical University.

### PDC Separation

Fresh peripheral blood mononuclear cells (PBMCs) were obtained from 76 healthy individuals. PDCs were isolated by magnetic-activated cell sorting using the Human Diamond Plasmacytoid Dendritic Cell Isolation Kit (Miltenyi Biotec). Purity of the isolated PDCs was >95%, as determined by flow cytometry (FCM).

### PDC Culture

Fresh human primary pDCs were cultured in complete RPMI 1640 medium (RPMI 1640 [Gibco, Invitrogen, Carlsbad, CA, USA] containing 10% fetal calf serum, 100 U/mL penicillin [Gibco], and 100 g/mL streptomycin [Gibco]). The cells were then exposed to 10^−4^ M glucocorticosteroid (Sigma) alone or with the TLR9 ligand (ODN2216, 5 µM) (Invivogen), a synthetic oligonucleotide containing unmethylated CpG motifs. Additionally, 20 ng/mL IL-3, which promotes pDC survival and activation, was added to the culture medium.

### miRNA Microarray

Total RNA was harvested using TRIzol (Invitrogen) and a miRNeasy mini kit (QIAGEN), according to the manufacturers’ instructions. After measuring RNA concentrations using a NanoDrop 1000, the samples were labeled using the miRCURY™ Hy3™/Hy5™ Power labeling kit and hybridized onto the miRCURY™ LNA Array (v.16.0). After washing, the slides were scanned using the Axon GenePix 4000B microarray scanner. The scanned images were then imported into GenePix Pro 6.0 software (Axon) for grid alignment and data extraction. Duplicate miRNAs were averaged, and miRNAs with intensities ≥50 were chosen to calculate the normalization factor. Expression data were normalized using median normalization. After normalization, significant differences in the expression of miRNAs were identified via T-test filtering.

### miRNA and siRNA Transfection of pDCs

miRNA mimics were synthesized by Ribobio (Guangzhou, China). siRNA for human MCL-1 was obtained from Santa Cruz (sc-35877). pDCs were transfected according to a previously described protocol [7]. FCM using FAM-tagged scramble siRNA was used to assess the transfection efficiency, and qPCR was used to determine the overexpression and knockdown efficacy of miRNA after transfection.

### PDC Apoptosis Analysis

PDCs were stained with annexin V-FITC/PI (Promega), and fluorescence was analyzed by FCM.

### Quantitative Real-time RT-PCR

Total cellular RNA was isolated using TRIzol (Invitrogen). cDNA was prepared by reverse transcription (PrimeScript RT Reagent Kit, Takara, Shiga, Japan) and amplified by real-time quantitative PCR (qPCR) with the primers shown in Table S1. Equal amounts of cDNA were used for the subsequent qPCR reactions, which were performed using the SYBR® PrimeScript® RT-PCR kit (Takara). Amplification was performed in an ABI PRISM 7900 Real-Time PCR System (Applied Biosystems). The amplification efficiency of the genes was the same as that of Rpl13a, as indicated by the standard curves for amplification, which allowed us to use the following formula: fold difference = 2^–(ΔCtA – ΔCtB)^, where Ct is the cycle threshold. miRNA expression was quantified using the TaqMan MicroRNA Expression Assay (Applied Biosystems), according to the manufacturer’s protocol. miRNA expression was normalized to that of endogenous RNU48.

### Western Blotting

Cells were lysed in RIPA Lysis and Extraction Buffer (Thermo Fisher Scientific Inc., Rockford, USA). Equal sample volumes were loaded into the wells of a 10% polyacrylamide-SDS gel, and the gels were run according to a standard protocol. The separated proteins were then transferred to an Immobilon-P polyvinylidene difluoride membrane (Millipore, Billerica, MA) via a standard wet-transfer protocol. The membrane was blocked with SuperBlock T20 PBS Blocking Buffer (Thermo Fisher Scientific Inc.), cut, and probed with the indicated primary antibodies. Then, the membranes were incubated with the appropriate horseradish peroxidase (HRP)-conjugated secondary antibody. The resulting bands were visualized using Pierce ECL Western Blotting Substrate (Thermo Fisher Scientific, Inc.), exposed to film, and digitized. MCL-1 was detected using a rabbit polyclonal antibody (sc-819; Santa Cruz Biotechnology Inc., CA, USA); Bcl-2 was detected using a goat polyclonal antibody (sc-492; Santa Cruz Biotechnology, Inc.); and β-actin was detected using a rabbit polyclonal antibody (#4970; Cell Signaling Technology, Danvers, Massachusetts, USA). The Bcl-xL antibody (#2762) and HRP-conjugated secondary antibodies were obtained from Cell Signaling Technology. The relative intensity of each band was quantified using image J 1.43 software.

### Data Analysis

miRNA targets were predicted using TargetScan Human Release 5.2 (http://www.targets can.org/vert_50/) [11] and RNA hybrid (http://bibiserv.techfak.uni-bielefeld.de/rnahybrid/submission.htm) software [12]. Data are expressed as the mean ± SD. All data were analyzed using a one-way analysis of variance (ANOVA). Multiple comparisons between groups were performed using the S-N-K method. A p value of less than 0.05 was considered statistically significant. Statistical analysis was carried out using StatView 5.0 software (SAS Institute, Cary, NC).

## Results

### miRNAs are Involved in CpG-inhibited Dex-induced pDC Apoptosis

**Figure 1:**
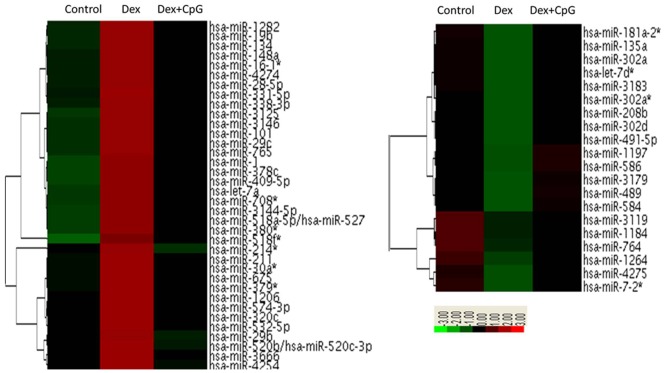
miRNAs are involved in CpG-inhibited Dex-induced pDC apoptosis. miRNA array analysis of miRNA expression in pDCs after stimulation with Dex alone or in combination with CpG. The expression levels of miRNAs that were altered by greater than a single fold are presented.

To investigate the involvement of miRNAs in TLR-inhibited GC-induced pDC apoptosis, human primary pDCs were treated with Dex alone or the combination of Dex and CpG for 16 hours. miRNA profiles were detected using miRNA microarrays (Figure 1). A total of 120 miRNAs were induced by Dex, including many known pro-apoptotic miRNAs, such as those of the let-7 family, miR-16, -23a, -31, -98, and -101, the miR-29 family, the miR-30 family, and the miR-320 family, among others. Further analysis showed that 36 of the 120 miRNAs were inhibited by CpG stimulation, which may have been due to CpG-inhibited GC-induced apoptosis (Table S2). However, 83 miRNAs were inhibited by Dex, and among these, 20 miRNAs were induced by CpG stimulation. In total, 56 miRNAs were involved in TLR-inhibited GC-induced apoptosis of human primary pDCs.

**Figure 2:**
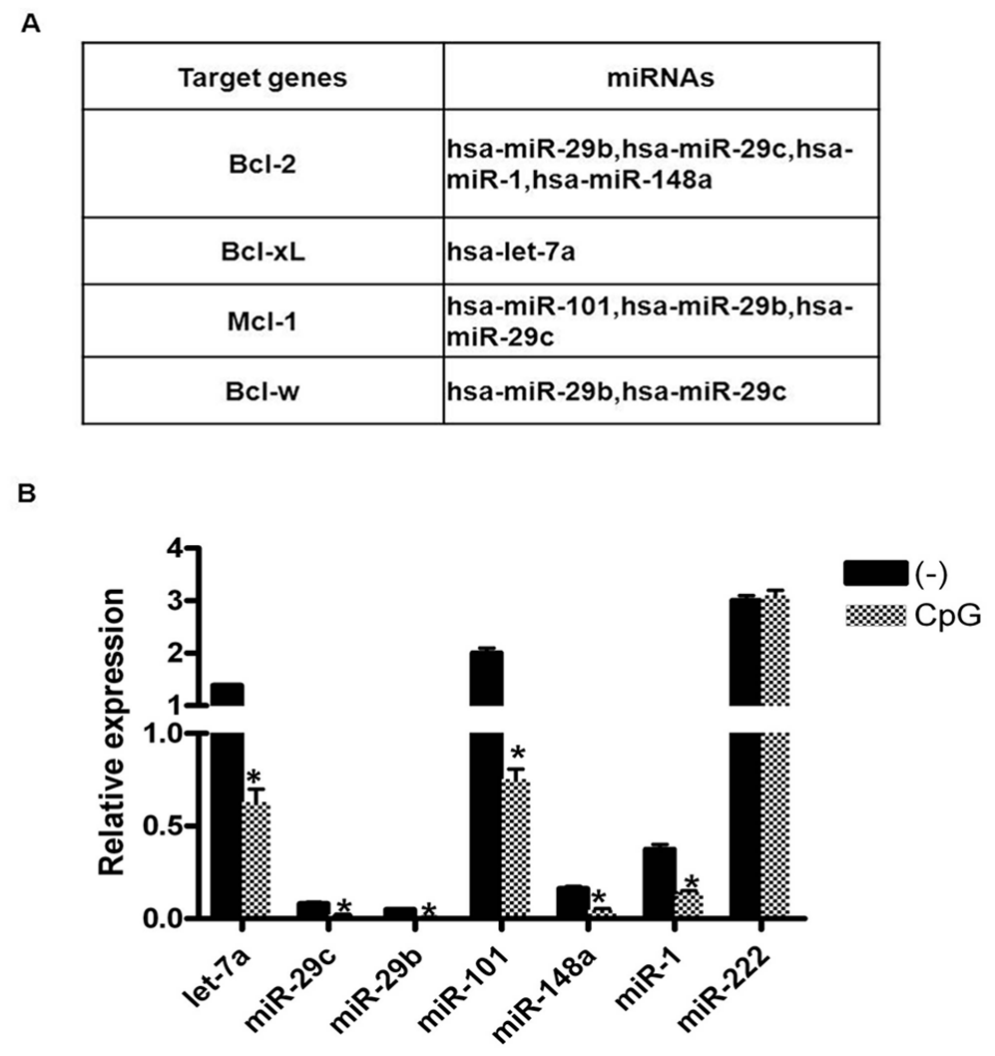
miRNAs predicted to target Bcl-2 family members. The miRNAs presented in Figure 1 were analyzed using bioinformatics. (A) miRNAs predicted to target Bcl-2 family members. (B) miRNA expression changes upon CpG stimulation. After 6 hours of CpG stimulation, pDC miRNAs were extracted and analyzed using a TaqMan MicroRNA Expression Assay. Expression levels were normalized to RNU48. The data are representative of at least 3 independent experiments, each using a different pDC preparation. Data are expressed as the mean ± SD and were analyzed with a 2-tailed Student’s t test. *P<0.05.

To identify functional miRNAs that regulate pDC apoptosis, 56 miRNAs were analyzed using bioinformatics. Target prediction analysis identified 6 miRNAs that could bind Bcl-2 family members (Figure 2A), and qRT-PCR further verified that these miRNAs were present and could be inhibited by CpG stimulation alone. miR-222, as a negative control, did not decrease in pDCs during CpG stimulation (Figure 2B). What’s more, 6 miRNAs (miR-let-7a, -29c, -29b, -101, -148a, and -1) were up-regulated following Dex stimulation, while CpG prevented their induction (Figure S1).These results indicate that the identified miRNAs may be important for the anti-apoptotic effect of TLR stimulation in human primary pDCs during culture with GCs.

### Mcl-1 is Indispensable for pDC Survival

The Bcl-2 family governs mitochondrial outer membrane permeabilization (MOMP), and its members can be either pro-apoptotic (e.g., Bax, BAD, Bak, and Bim) or anti-apoptotic (e.g., Bcl-2, Bcl-xL, Mcl-1, and Bcl-w) [13]. In a previous study, the inhibition of Bcl-2 and c-flip dramatically decreased pDC viability during CpG culture alone or with Dex, whereas inhibition of Bcl-xL and birc-3/iap-2 did not significantly alter pDC survival [7].

**Figure 3:**
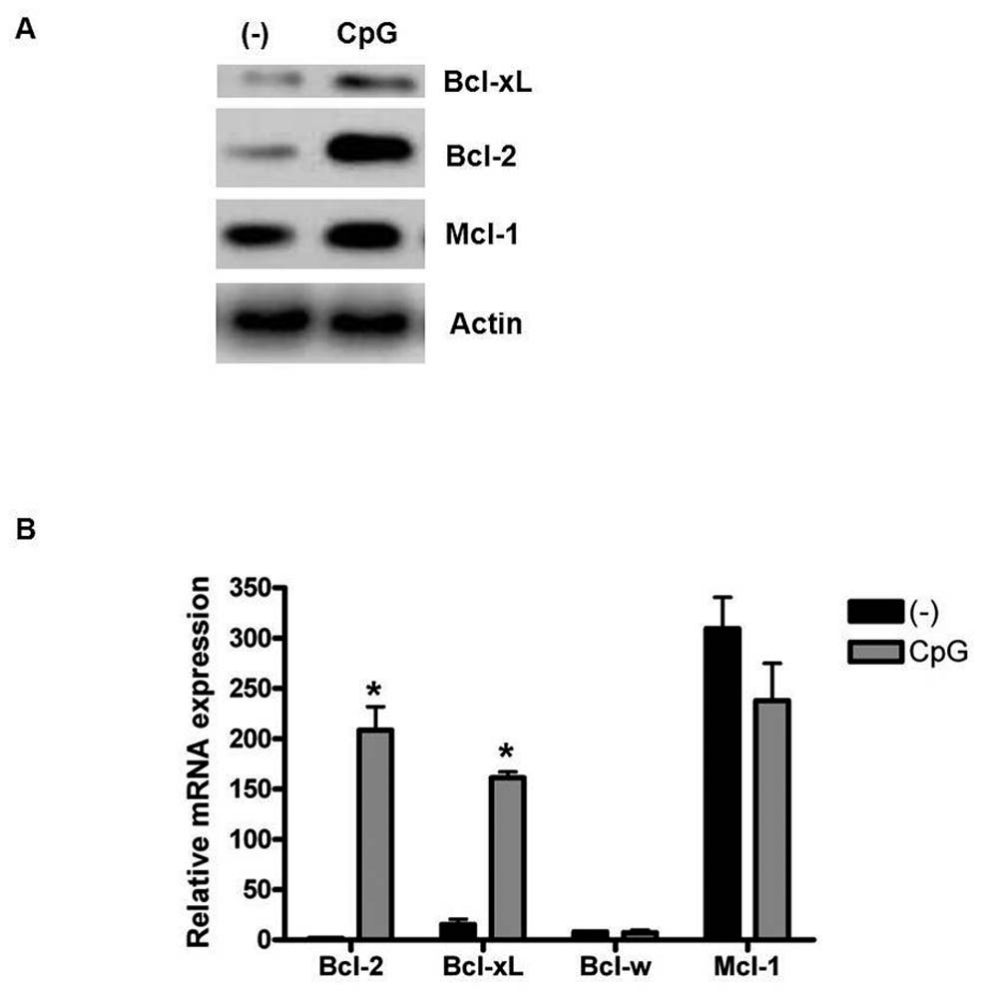
Changes in Bcl-2 family member expression in human primary pDCs following CpG stimulation. Western blot (A) and qRT-PCR (B) analyses of Bcl-xL, Mcl-1, and Bcl-2 expression after 12 hours of stimulation with CpG. The data are representative of at least 3 independent experiments, each using a different pDC preparation. Data are expressed as the mean ± SD and were analyzed with a 2-tailed Student’s t test. *P<0.05.

**Figure 4:**
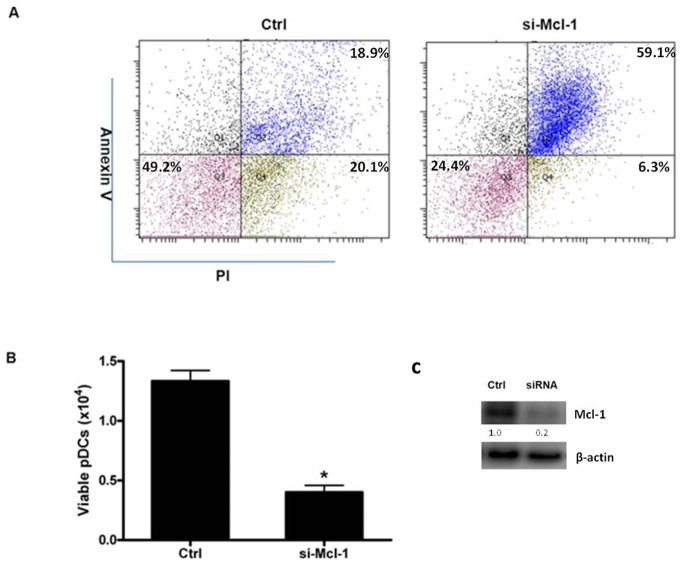
Mcl-1 is indispensable for pDC survival. (A, B) Primary pDCs were transfected with control (Ctrl) or Mcl-1 siRNA, and pDC apoptosis was analyzed by FCM. (C) siRNA-mediated knockdown of Mcl-1 expression was assessed by western blot analysis. The data are representative of at least 3 independent experiments, each using a different pDC preparation. Data are expressed as the mean ± SD and were analyzed with a 2-tailed Student’s t test. *P<0.05.

Although the functions of Bcl-2 and Bcl-xL in TLR-inhibited GC-induced pDC apoptosis have been studied, the role of other anti-apoptotic members, which are predicted targets of the selected 6 miRNAs, is not well understood. To obtain a more comprehensive understanding of the function of anti-apoptotic members of the Bcl-2 family in this process, we first evaluated the expression levels of Bcl-2, Bcl-xL, Mcl-1, and Bcl-w in pDCs (Figure 3A). We did not address the role of A1, as it was not a predicted target of the altered miRNAs. Mcl-1 was the only member of the Bcl-2 family that was abundantly expressed in fresh pDCs. Upon stimulation, the mRNA and protein levels of Bcl-2 and Bcl-xL increased rapidly, which is consistent with a previous report (Figure 3A and B) [7]. In contrast, the Mcl-1 mRNA level was unchanged, although its protein level increased, indicative of post-transcriptional regulation of Mcl-1 expression. Bcl-w was consistently expressed at a low level (Figure 3B). Because Bcl-xL has no effect on pDC apoptosis, we evaluated whether Mcl-1 expression may have a functional role in this process. As Mcl-1 expression decreased, pDC apoptosis was observed (Figure 4A–C), which suggests that Mcl-1 is indispensable for pDC survival and that, similar to Bcl-2, Mcl-1 is functional during this process.

To exclude adverse effects of Mcl-1 knockdown on the other Bcl-2 family members, we evaluated Bcl-2 expression in Mcl-1 siRNA-transfected samples after CpG stimulation, and knockdown of Mcl-1 had no effect on Bcl-2 expression (Figure S2). The results demonstrated that Mcl-1 has a specific role of modulating pDC apoptosis. Then we evaluated the function of Mcl-1 during CpG stimulation, and Mcl-1 knockdown also enhanced apoptosis in CpG-treated cells, albeit to a lesser extent (Figure S3). All these results indicate that Mcl-1 plays an important role in pDC survival.

### miR-29b and miR-29c Rescue Dex-induced pDC Apoptosis during CpG Stimulation

**Figure 5:**
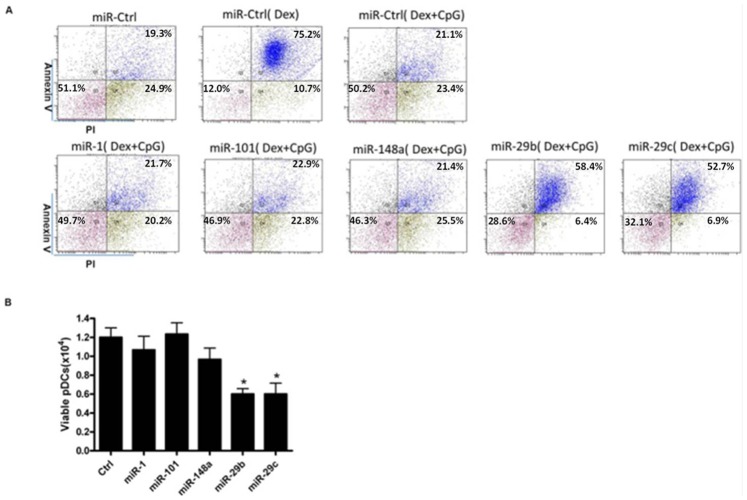
miR-29b and miR-29c are involved in TLR-inhibited Dex-induced pDC apoptosis. (A, B) Primary pDCs were transfected with control (Ctrl), miR-1, miR-101, miR-148, miR-29b, or miR-29c mimics. Then, cells were stimulated with Dex alone or the combination of Dex and CpG, and pDC apoptosis was analyzed 16 hours post-stimulation. The data are representative of at least 3 independent experiments, each using a different pDC preparation. Data are expressed as the mean ± SD and were analyzed with a 2-tailed Student’s t test. *P<0.05.

To test the function of the selected miRNAs, pDCs were transfected with miRNA mimics and cultured with GCs alone or in combination with CpG. First, we assessed the transfection efficiency of miRNAs mimics, miRNA inhibitors, and FAM-tagged scramble RNA by FCM and qRT-PCR. Transfection of miR-29 enhanced its expression level by approximately 16-fold in comparison to the control group, whereas transfection of the miR-29 inhibitor dramatically reduced its expression level (Figure S4). In accordance with previous reports, TLR9 stimulation greatly enhanced pDC survival in the presence of Dex, and overexpression of miR-29b/c promoted pDC apoptosis, indicating that these miRNAs play important roles during this process (Figure 5A and B). However, other miRNAs that were also predicted to directly target Bcl-2 family members had no effect on pDC apoptosis.

To verify the effect of miR-29b and miR-29c in pDC apoptosis during Dex stimulation alone, we transfected miR-29b or miR-29c inhibitors to verify their effect on pDC apoptosis after stimulation with Dex alone. The inhibition of miR-29b and miR-29c was shown to promote pDC survival (Figure S5). These results further indicated that miR-29b and miR-29c are involved in pDC apoptosis.

### miR-29b and miR-29c Promote pDC Apoptosis by Targeting Mcl-1 and Bcl-2

**Figure 6:**
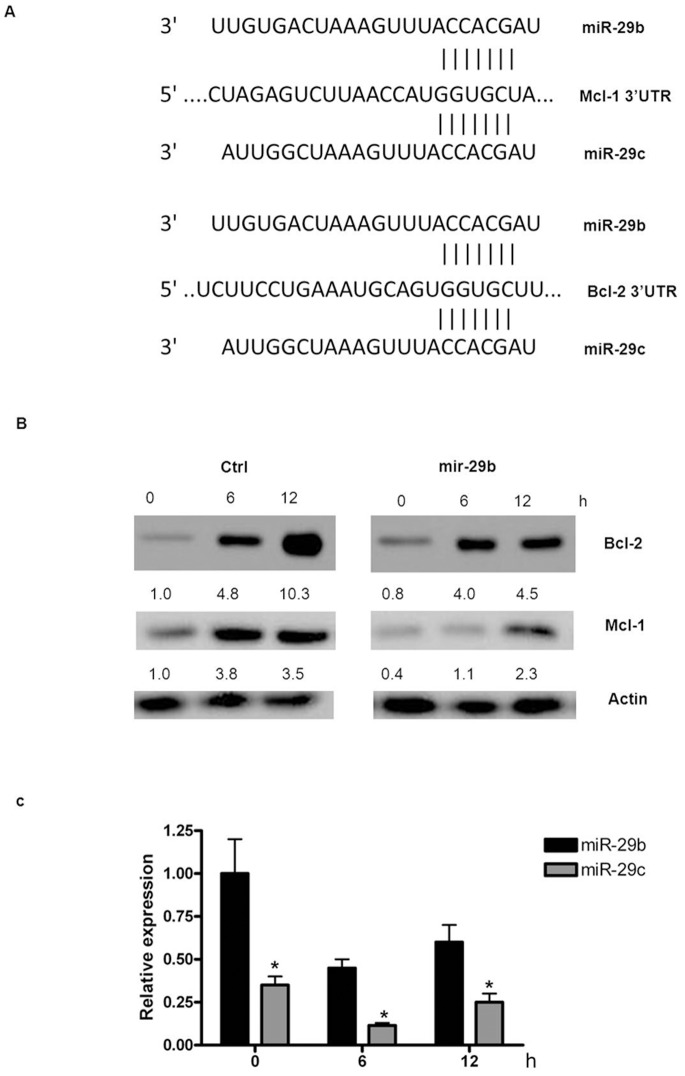
miR-29 promotes pDC apoptosis by targeting Mcl-1 and Bcl-2. (A) Target prediction. (B) miR-29 inhibited Mcl-1 and Bcl-2 expression. Primary pDCs were transfected with control (Ctrl) or miR-29b mimics and stimulated with CpG. The protein levels of Mcl-1 and Bcl-2 were analyzed by western blot. (C) Kinetics of miR-29b and miR-29c during CpG stimulation. The data are representative of at least 3 independent experiments, each using a different pDC preparation. Data are expressed as the mean ± SD and were analyzed with a 2-tailed Student’s t test. *P<0.05.

Previous reports have demonstrated that both Mcl-1 and Bcl-2 are inhibited by miR-29 in various cell types [14], [15]. We also found that the 3′-UTRs of Mcl-1 and Bcl-2 were targets of miR-29 (Figure 6A). To determine whether miR-29 would demonstrate a similar function in human primary pDCs, cells were transfected with miR-29 mimics, stimulated with CpG, and the protein levels of Bcl-2 and Mcl-1 were evaluated. The results indicated that miR-29 overexpression inhibited both Bcl-2 and Mcl-1 expression (Figure 6B).

To further verify the relationship between miR-29b/c and their targets, we evaluated their expression kinetics. Both miR-29b and miR-29c were inhibited by TLR9 stimulation (Figure 6C), and as their miRNA levels decreased, the protein levels of Mcl-1 and Bcl-2 increased. Considering that the mRNA level of Mcl-1 did not increase during CpG stimulation, the increased protein expression observed further indicates that Mcl-1 is post-transcriptionally regulated by miRNAs.

## Discussion

Several reports have demonstrated that all 3 miR-29 paralogs are expressed at increased or decreased levels in different types of cancers in comparison to corresponding normal tissues [16]–[18]. Notably, miR-29 has been hypothesized to positively associate with p53 to induce apoptosis. Moreover, miR-29 targets Mcl-1 in human hepatocellular carcinoma cells and acute myelogenous leukemia cells, and its expression is down-regulated in both types of cancer [14]. In this study, we demonstrated that down-regulation of miR-29 was correlated with drug resistance in pDCs, supporting the idea that miR-29 is a pro-apoptotic factor during this process.

Although miR-29b and miR-29c show relative low expression levels in fresh pDCs, they could be induced to relatively medium levels during Dex stimulation (Table S3).Our study further found that miR-29b and miR-29c induced less apoptosis in comparison to Dex treatment alone (Figure 5). As other molecules may also be involved in Dex-induced pDC apoptosis, overexpression of miR-29b or miR-29c could not completely eliminate the inhibitory effect of CpG on Dex-induced apoptosis.

Mcl-1, the target of miR-29b, was the only member of the Bcl-2 family that was abundantly expressed in fresh pDCs. Upon stimulation, the mRNA and protein levels of Bcl-2 and Bcl-xL increased rapidly, which is consistent with a previous report (Figure 3A and B) [7]. In contrast, the Mcl-1 mRNA level was unchanged, although its protein level increased, indicative of post-transcriptional regulation of Mcl-1 expression.

The regulation of miR-29 expression has been previously studied, and NFκB was shown to negatively regulate miR-29 expression [19], [20]. In our study, we found that the expression of miR-29b and miR-29c were both inhibited by TLR9 stimulation. Considering that the NFκB pathway is activated by TLR9 in human primary pDCs, it is reasonable to hypothesize that TLR-inhibited miR-29 expression facilitates the function of NFκB in alleviating Dex-induced pDC apoptosis.

The post-transcriptional regulation of Bcl-2 members by different miRNAs has been extensively studied [9]. Similarly, we found many Dex-induced miRNAs that directly targeted Bcl-2, Mcl-1, and Bcl-xL. For example, miR-16, which was shown to negatively regulate Bcl-2 expression [21], [22], was induced by Dex treatment (Table S3). However, there was no difference in the expression of miR-16 between the Dex- and Dex+CpG-treated samples, indicating that TLR stimulation does not function through miR-16; instead, our results indicated that miR-29b and miR-29c may participate in pDC apoptosis. These results suggest that miRNA functions may be specific to the cell type and cellular process under investigation.

The presence of anti-apoptotic pDCs in SLE is thought to explain the high IFN-related gene expression and resistance to treatment [6]. Therefore, compounds that block TLR activation have been proposed as adjuvants to improve the effects of GC treatment. In this study, we proposed that miRNAs may provide a new treatment for autoimmune diseases. In support of this notion, miR-29-deficient mice were shown to have significantly greater numbers of Th1 cells and increased IFN-γ production [20]. However, further examination of the expression levels of miR-29 family members in pDCs from SLE patients is necessary to determine whether such factors are abnormally expressed in the diseased state.

## Supporting Information

Figure S1 **qRT-PCR verification of changed miRNAs.** miRNAs were extracted and analyzed by TaqMan MicroRNA Expression Assay. Expression levels were normalized to RNU48. The data are representative of at least three independent experiments, each based on a different pDC preparation. Data are expressed as the mean ± SD and were analyzed with a 2-tailed Student’s t test. *P<0.05.
(TIF)

Figure S2 **Knock down of Mcl-1.** (A,B) Western blot analysis of knock-down effect of Mcl-1 siRNA on Mcl-1and Bcl-2 protein expression with or without CpG stimulation. The data are representative of three independent experiments, each based on a different pDC preparation. Data are expressed as the mean ± SD and were analyzed with a 2-tailed Student’s t test. *P<0.05.
(TIF)

Figure S3 **Mcl-1 maintains pDC survival during CpG stimulation.** (A,B) pDCs transfected with control (Ctrl) or Mcl-1 siRNA were treated with CpG. The apoptosis of pDCs were detected by FACS. The data are representative of three independent experiments, each based on a different pDC preparation. Data are expressed as the mean ± SD and were analyzed with a 2-tailed Student’s t test. *P<0.05.
(TIF)

Figure S4 **Transfection efficiency of siRNA.** (A,B)The Fam positive viabel pDCs were detected by FACS. (C) Overexpression or knock-down of miRNA in primary pDCs. pDCs were transfected with control (Ctrl), miR-29b mimic, miR-29c mimic, miR-29b inhibitor or miR-29c inhibitor. 24 hours after transfection, miRNA expression levels were detected. The data are representative of three independent experiments, each based on a different pDC preparation. Data are expressed as the mean ± SD and were analyzed with a 2-tailed Student’s t test. *P<0.05.
(TIF)

Figure S5 **Inhibition of miR-29b or miR-29c partly ameliorated Dex-induced pDC apoptosis.** (A,B) pDCs transfected with control (Ctrl), miR-29b inhibitor or miR-29c inhibitor were treated with Dex. The apoptosis of pDCs were detected by FACS. The data are representative of three independent experiments, each based on a different pDC preparation. Data are expressed as the mean ± SD and were analyzed with a 2-tailed Student’s t test. *P<0.05.
(TIF)

Table S1 **Primers for qRT-PCR.**
(DOC)

Table S2 **36 miRNAs were inhibited by CpG stimulation.**
(XLS)

Table S3 **miRNA array analysis of miRNA expression in pDCs after stimulation with Dex alone or in combination with CpG.**
(XLSX)
