# Supplementary figures and images for: miR-29b and miR-29c Are Involved in Toll-Like Receptor Control of Glucocorticoid-Induced Apoptosis in Human Plasmacytoid Dendritic Cells
Source: PLoS One. 2013 Jul 23;8(7):e69926. doi: 10.1371/journal.pone.0069926 (PMC3720938; doi:10.1371/journal.pone.0069926)

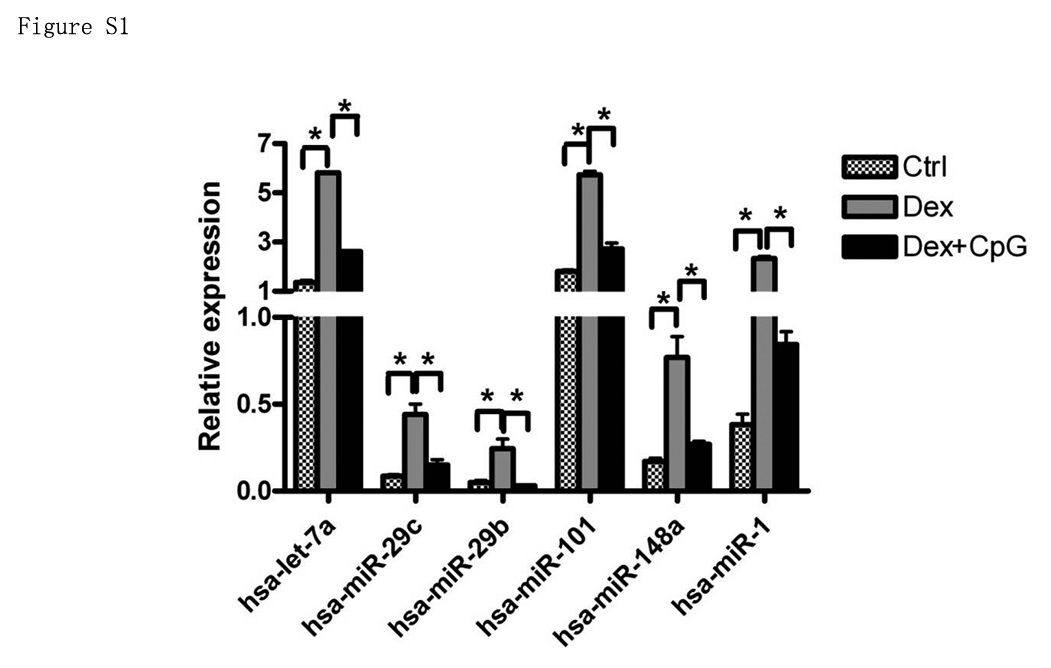

Supplement: Figure S1 — qRT-PCR verification of changed miRNAs. miRNAs were extracted and analyzed by TaqMan MicroRNA Expression Assay. Expression levels were normalized to RNU48. The data are representative of at least three independent experiments, each based on a different pDC preparation. Data are expressed as the mean ± SD and were analyzed with a 2-tailed Student’s t test. *P<0.05. (TIF) [file pone.0069926.s001.tif]

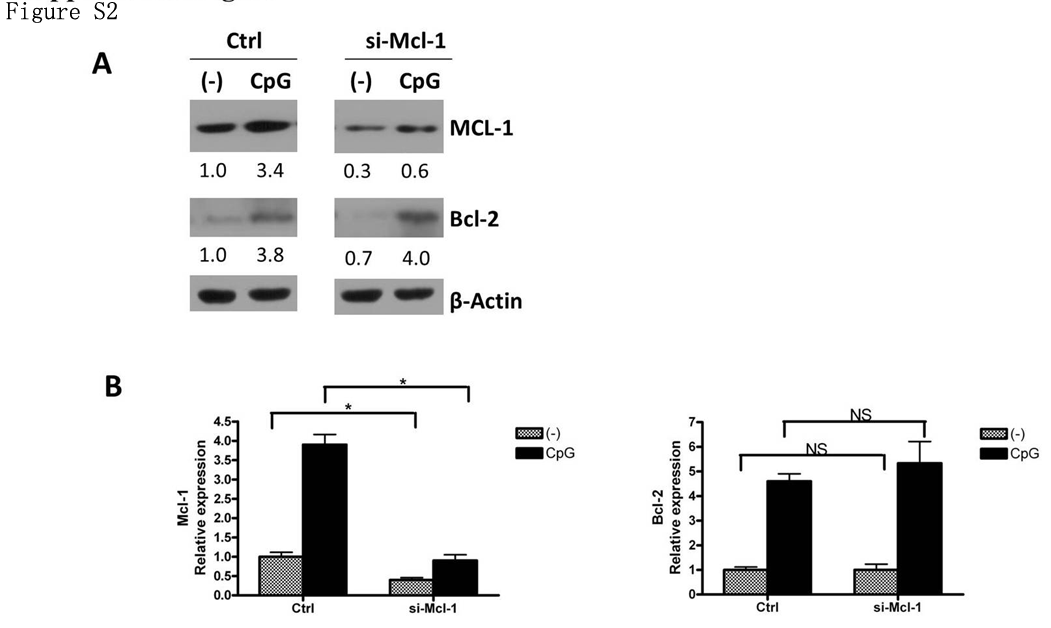

Supplement: Figure S2 — Knock down of Mcl-1. (A,B) Western blot analysis of knock-down effect of Mcl-1 siRNA on Mcl-1and Bcl-2 protein expression with or without CpG stimulation. The data are representative of three independent experiments, each based on a different pDC preparation. Data are expressed as the mean ± SD and were analyzed with a 2-tailed Student’s t test. *P<0.05. (TIF) [file pone.0069926.s002.tif]

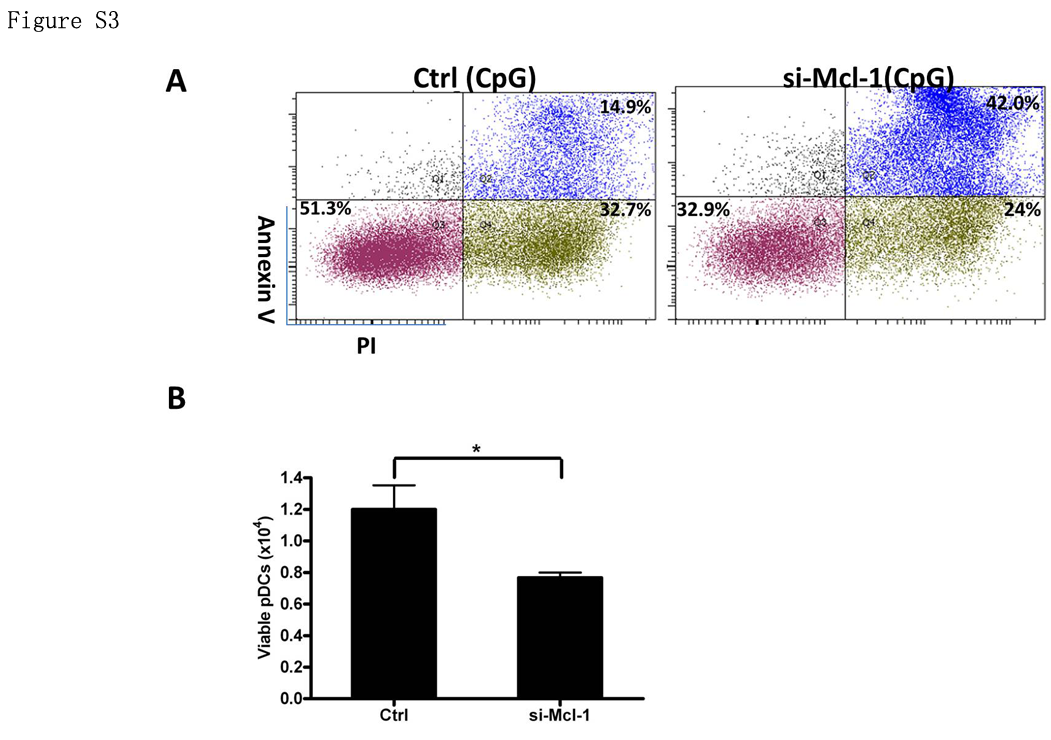

Supplement: Figure S3 — Mcl-1 maintains pDC survival during CpG stimulation. (A,B) pDCs transfected with control (Ctrl) or Mcl-1 siRNA were treated with CpG. The apoptosis of pDCs were detected by FACS. The data are representative of three independent experiments, each based on a different pDC preparation. Data are expressed as the mean ± SD and were analyzed with a 2-tailed Student’s t test. *P<0.05. (TIF) [file pone.0069926.s003.tif]

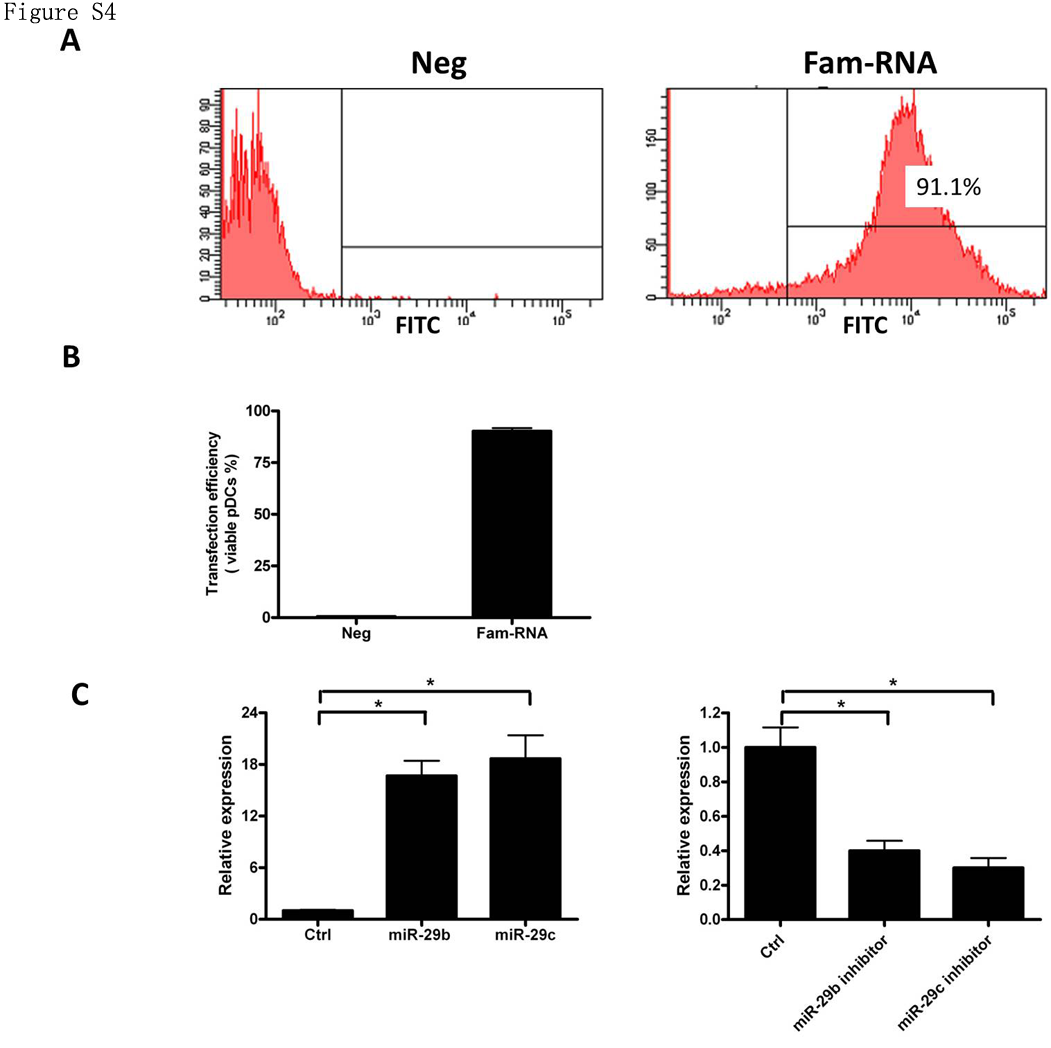

Supplement: Figure S4 — Transfection efficiency of siRNA. (A,B)The Fam positive viabel pDCs were detected by FACS. (C) Overexpression or knock-down of miRNA in primary pDCs. pDCs were transfected with control (Ctrl), miR-29b mimic, miR-29c mimic, miR-29b inhibitor or miR-29c inhibitor. 24 hours after transfection, miRNA expression levels were detected. The data are representative of three independent experiments, each based on a different pDC preparation. Data are expressed as the mean ± SD and were analyzed with a 2-tailed Student’s t test. *P<0.05. (TIF) [file pone.0069926.s004.tif]

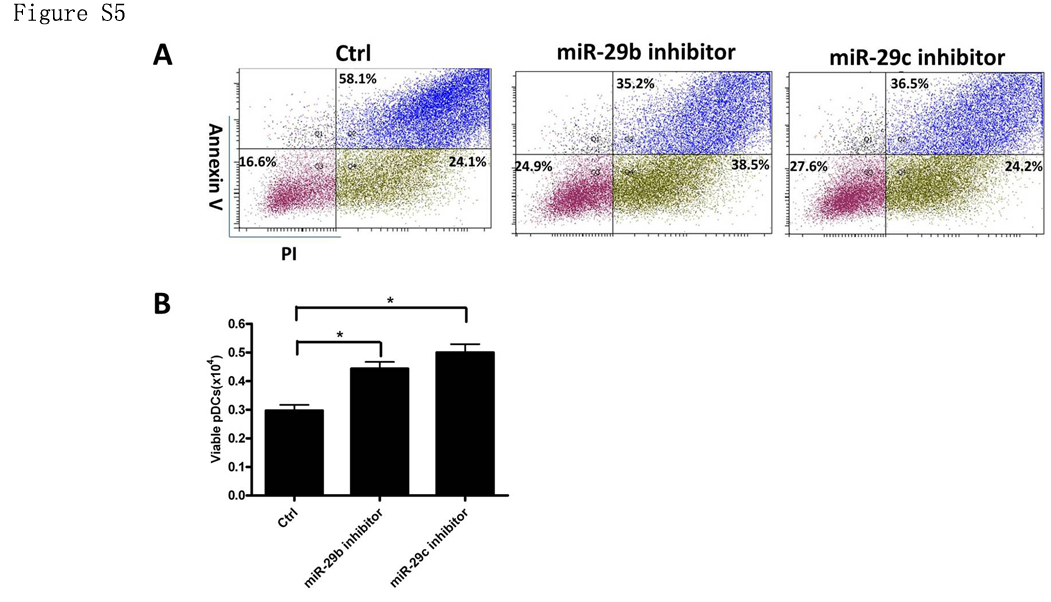

Supplement: Figure S5 — Inhibition of miR-29b or miR-29c partly ameliorated Dex-induced pDC apoptosis. (A,B) pDCs transfected with control (Ctrl), miR-29b inhibitor or miR-29c inhibitor were treated with Dex. The apoptosis of pDCs were detected by FACS. The data are representative of three independent experiments, each based on a different pDC preparation. Data are expressed as the mean ± SD and were analyzed with a 2-tailed Student’s t test. *P<0.05. (TIF) [file pone.0069926.s005.tif]
