# Supplementary material for: miR-29b and miR-29c Are Involved in Toll-Like Receptor Control of Glucocorticoid-Induced Apoptosis in Human Plasmacytoid Dendritic Cells
Source: PLoS One. 2013 Jul 23;8(7):e69926. doi: 10.1371/journal.pone.0069926 (PMC3720938; doi:10.1371/journal.pone.0069926)
Supplement: Table S1 — Primers for qRT-PCR. (DOC) [file pone.0069926.s006.doc]

**Table S1. Primers for qRT-PCR.**

| **Genes** | **Forward 5’-3’** | **Reverse 5’-3’** |
| --- | --- | --- |
| Mcl-1 | ATGCTTCGGAAACTGGACAT | TCCTGATGCCACCTTCT AGG |
| Bcl-2 | GGAGGATTGTGGCCTTCTTT | GGAGGATTGTGGCCTTCTTT |
| Bcl-w | ACCCCAGGCTCAGCCCAACA | CAGCACACAGTGCAGCCCCA |
| Bcl-xL | GGATGGC CACTTACCTGA | GCCGTACAGTTCCACAAAGG |
| Rpl13a | CCTGGAGGAGAAGAGGAAAGAGA | TTGAGGACCTCTGTGTATTTGTCAA |
